# S13-4: Using the socio-ecological model of physical activity to test the efficacy of a social intervention at an active leisure event: the parkwalker initiative

**DOI:** 10.1093/eurpub/ckae114.258

**Published:** 2024-09-26

**Authors:** Andre Gilburn

**Affiliations:** University of Stirling, Stirling, Scotland

## Abstract

**Purpose:**

The socio-ecological model of participation in physical exercise assumes that individual and environmental components interact to shape patterns of activity. The model proposes that the environmental component can be broadly separated into the physical, social and political environments. The active leisure event organiser parkrun recently introduced a new volunteer role, the parkwalker, to manipulate the social environment at their events to encourage more walkers to attend. The aim of this study was to determine the success of the parkwalker initiative.

**Methods:**

This study builds a socio-ecological model of the finishing times of new parkrun participants in Scotland for a year before and after the introduction of the parkwalker initiative to determine if the intervention has worked. Event locations in Scotland were separated into those that fully adopted, partially adopted and did not adopt the parkwalker role into their events.

**Results:**

The model of finishing times revealed they have slowed after the introduction of parkwalkers and the level of slowing is associated with the level of adoption of the role by events. The parkwalker initiative was particularly effective on the impact of finishing times of older new participants and at larger events. The initiative was also associated with an increase in the proportion of female new participants and a reverse in the recent decline in the age of new participants.

**Conclusions:**

This suggests that parkrun have introduced a successful intervention to their events that has manipulated the social environment to increase both engagement and inclusivity. This has management implications for both parkrun and other active leisure events as the study reveals it is possible to successfully manipulate the social environment of events to increase engagement. The study also highlights how the socio-ecological model can be used to test the efficacy of an intervention and quantify and understand its impact.

**Funding:**

None

